# Muscle Fatigue in the Three Heads of the Triceps Brachii During a Controlled Forceful Hand Grip Task with Full Elbow Extension Using Surface Electromyography

**DOI:** 10.1515/hukin-2015-0035

**Published:** 2015-07-10

**Authors:** Asraf Ali, Kenneth Sundaraj, R. Badlishah Ahmad, Nizam Uddin Ahamed, Anamul Islam, Sebastian Sundaraj

**Affiliations:** 1 AI-Rehab Research Group, Universiti Malaysia Perlis (UniMAP), Kampus Pauh Putra, Perlis, Malaysia.; 2 Mechatronic Engineering, Faculty of Manufacturing Engineering, Universiti Malaysia Pahang,Pekan, Malaysia.; 3 Medical Officer, Malaysian Ministry of Health, Malaysia.

**Keywords:** isometric contraction, muscle fatigue, surface electromyography, triceps brachii muscle

## Abstract

The objective of the present study was to investigate the time to fatigue and compare the fatiguing condition among the three heads of the triceps brachii muscle using surface electromyography during an isometric contraction of a controlled forceful hand grip task with full elbow extension. Eighteen healthy subjects concurrently performed a single 90 s isometric contraction of a controlled forceful hand grip task and full elbow extension. Surface electromyographic signals from the lateral, long and medial heads of the triceps brachii muscle were recorded during the task for each subject. The changes in muscle activity among the three heads of triceps brachii were measured by the root mean square values for every 5 s period throughout the total contraction period. The root mean square values were then analysed to determine the fatiguing condition for the heads of triceps brachii muscle. Muscle fatigue in the long, lateral, and medial heads of the triceps brachii started at 40 s, 50 s, and 65 s during the prolonged contraction, respectively. The highest fatiguing rate was observed in the long head (slope = −2.863), followed by the medial head (slope = −2.412) and the lateral head (slope = −1.877) of the triceps brachii muscle. The results of the present study concurs with previous findings that the three heads of the triceps brachii muscle do not work as a single unit, and the fiber type/composition is different among the three heads.

## Introduction

Muscle fatigue is defined as a lack of ability to generate force by a muscle or a group of muscles (Gandevia, 2001; Edwards, 1981). Muscle fatigue during voluntary contraction is a multifactor phenomenon that occurs in the central nervous system as well as the peripheral site of the neuromuscular system (Bigland-Ritchie et al., 1978). However, muscle fatigue may develop due to failure at one or several locations along the pathway of force production as a result of performing any type of muscular work. A number of methods exist to quantify muscle fatigue in humans during muscular work. For example, the study of Ángyán et al. (2007) and Emery and Côté (2012) examined movement accuracy and found that repeated tasks induced muscle fatigue. In contrast, duration for which a task can be sustained at a given level of maximal voluntary contraction has been widely used to quantify muscle fatigue and is termed as the endurance time or time to task failure (West et al., 1995). Many individual factors can influence muscle fatigue, and it has previously been shown that the individual strength level is a major determinant factor during a fatiguing task (Hunter et al., 2004). The development and analysis of muscle fatigue have been studied by the non-invasive technique of surface electromyography (sEMG). For example, the studies by De Luca (1984) and Masuda et al. (1999) noted that sEMG was able to monitor the fatigue process in different muscles simultaneously. As far as upper limb muscles are concerned, previous studies have shown that fatigue can alter these muscle activation patterns in single or multi-joint tasks, such as fast elbow flexion (Corcos et al., 2002), elbow extension (Popadic Gacesa et al., 2010), swimming (Ikuta et al., 2012; Stirn et al., 2011), rowing (Gerževic et al., 2011), and concurrent elbow flexion/extension with a forceful hand grip exercise (Oda and Kida, 2001).

It is generally agreed that muscle fatigue is documented based on an amplitude variable in the time domain or a frequency variable in a frequency domain. For example, the study of Alizadehkhaiyat et al. (2011) analysed the time-slope of the root mean square of the amplitude and median frequency to measure the fatigability of two key shoulder girdle muscles, namely the supraspinatus and infraspinatus, during a gripping task. On the other hand, the study by Blackwell et al. (1999) analysed the median power frequency of the electromyoghaphic (EMG) amplitude to investigate the effect of fatigue of the flexor digitorum superficialis muscle during sustained voluntary contractions at 60–65% of the maximum voluntary contraction of the forceful hand grip. Another study by Oda and Kida (2001) investigated muscle fatigue using the root mean square of the amplitude in the time domain during a sustained maximal isometric contraction for a concurrent forceful hand grip and elbow flexion/extension on the following upper limb muscles: flexor digitorum superficialis, extensor digitorum, biceps brachii and lateral head of the triceps brachii.

Triceps brachii (TB) is one of the principal and longest muscle of the upper limb which is generally acknowledged as an extensor of the forearm across the elbow joint (Moore and Dalley, 1992). However, it is known that the three heads of the TB do not work as a single unit throughout an extension movement (Landin and Thompson, 2011). Furthermore, it has been observed that static contractions at even very low force levels were found to cause spectral compression in the sEMG signals from the TB (Fallentin et al., 1985). These observations have motivated us to consider looking into muscular fatigue in the TB, more precisely among the three heads of the TB. Therefore, the aim of the present study was to investigate fatiguing conditions of the TB muscle by comparing the time to fatigue and fatiguing rates at the three heads (long, lateral, and medial), respectively. To investigate these parameters, we chose to execute two tasks concurrently to produce isometric contractions in the TB muscle – full elbow extension and a forceful hand grip. While the chosen exercise is not a common training position, the tasks involved (simultaneous full elbow extension with a forceful grip task) is very important in some sports like gymnastics.

## Material and Methods

### Subjects

Eighteen healthy male subjects between the ages of 21 and 27 years participated in this study. The mean and standard deviations of the characteristics of the participants were the following: age = 23.4 ± 2.3 years, body height = 172.1 ± 4.8 cm, body mass = 72.6 ± 6.1 kg. Subjects with a history of shoulder, elbow, or wrist injury were excluded from the study. Prior to participation in this study, all of the subjects read and signed an informed consent form approved by an Independent Ethics Committee (IEC).

### Experimental protocol

The subjects sat erect on a rigid chair furnished with an approximately vertical backrest. Additionally, they were asked to maintain their arm at the position of shoulder abduction with the scapula fixed at 90° abducted and the palm facing down towards the ground during the task. We selected this arm position because it is the neutral position of the upper limb between abduction and adduction of the shoulder. To reduce the influence of any other parts of the body activity on the TB contractions, the subjects were asked to maintain their posture during the task without any trunk movement. Prior to fatigue experiments, a maximal forceful hand grip test was measured by a digital hand grip dynamometer (Digital Hand Dynamometer, DHD-1, SAEHAN Corporation, South Korea) in a standardized position. Subjects were verbally instructed to produce the highest hand grip force for 5 s with full elbow extension for the measurement of maximal voluntary contraction (MVC). This exercise was performed three times (3 trials), where subjects were asked to take a 2 min rest between trials to minimize the potential effect of muscle fatigue. The average value from these three measurements was considered as the MVC. After a 10 min rest, the subjects were asked to perform a single hand grip with force at 80% MVC level for 90 s with full elbow extension for fatigue experiment. Trials were excluded when the hand grip force deviated above ±5 kg of the 80% MVC level. In case of trial exclusion, subjects repeated the task after a 10 min rest until a successful trial was performed.

### sEMG recording

**Figure 1:**
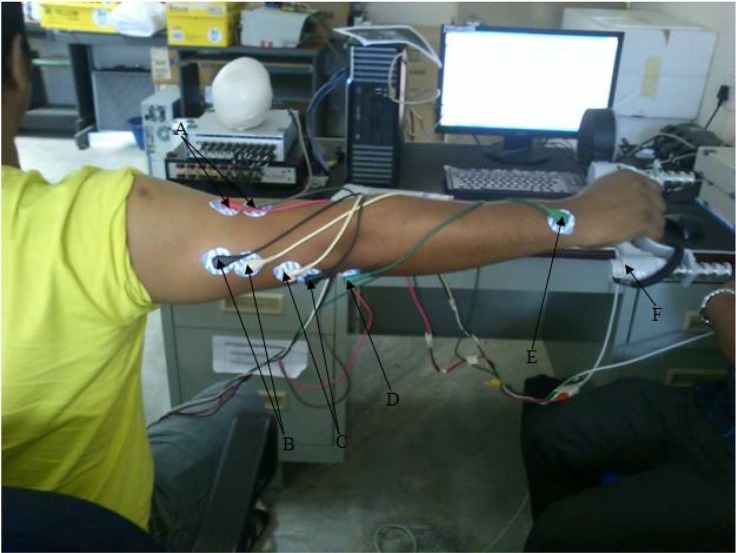
Electrode placement on the three heads of TB muscle (A=Lateral head, B=Long head, C=Medial head, D=Proximal head of ulna, E=Distal head of ulna, F=Hand grip dynamometer)

For the sEMG signal recording, we used two PowerLab systems (Model 4/25T by ADInstruments Pty. Ltd., Bella Vista, NSW, Australia) where each system consists of two channels with a common isolated ground. These two PowerLab systems were interfaced to a computer using the LabChart® (Version 7 by ADInstruments Pty. Ltd., Bella Vista, NSW, Australia) software for Windows®. Appropriate skin areas of the TB were shaved, cleaned with alcohol, and abraded with emery paper. We placed and aligned the disposable pregelled bipolar Ag/AgCl surface electrodes (Kendall Meditrace^TM^ 100 Tyco Healthcare Group LP, Mansfield, MA, USA) to the muscle fibre direction in the lateral, long, and medial heads of the TB, where the inter-electrode distance was 2.5 cm. The references electrodes were placed to the proximal head of ulna, and the distal head of ulna of the same arm. Electrodes placements are anatomically indicated in Figure 1. All muscle identification and electrode placement were observed and validated by a medical personnel present during the experimentation. The sEMG signals were recorded at a bandwidth of 10 to 500 Hz, using a differential amplifier (BioAmp, ADInstruments, Australia), A/D converted at 1000 Hz (16 bit resolution) and stored in a computer for further analysis. Once the sEMG amplitudes (in mV) from the three heads of the TB were recorded, sEMG data were digitally filtered (bandpassed between 20 to 250 Hz) to reduce motion and electrocardiopraphic artefacts. Before the actual trial, the MVC – RMS was calculated from the average of the RMS values taken during the three MVC trials. During the actual trials, the RMS values were then calculated for every 5 s throughout the isometric contraction of 90 s for each subject (1 value per 5 s segment per subject per head of TB). The RMS value for each 5 s segment of sEMG signals was then normalized with respect to the MVC – RMS value for each subject and expressed as a percentage.

### Statistical analysis

Descriptive statistics including the mean, standard deviation (SD) and the coefficient of variance in percentage (CV% which is the SD expressed as a percentage of the mean) of the normalized RMS values for each 5 s period of the total 90 s isometric contraction period of 18 subjects were calculated for the three heads of TB. A paired t-test was applied for the comparison of the normalized RMS values between the initial period (1–5 s) and the following periods, where variations with a probability level of p < 0.05 were considered as significant. The mean values for each period (5 s) were expressed as percentages of the initial period (1) value for the long, lateral, and medial heads of the TB. Then, a linear regression function was applied to these values to determine and compare the fatiguing rate among the three heads of the TB. All of the statistical calculations were executed using the SPSS 10.0 for Windows® (SPSS Inc., Chicago, IL, USA) statistical package.

## Results

**Figure 2:**
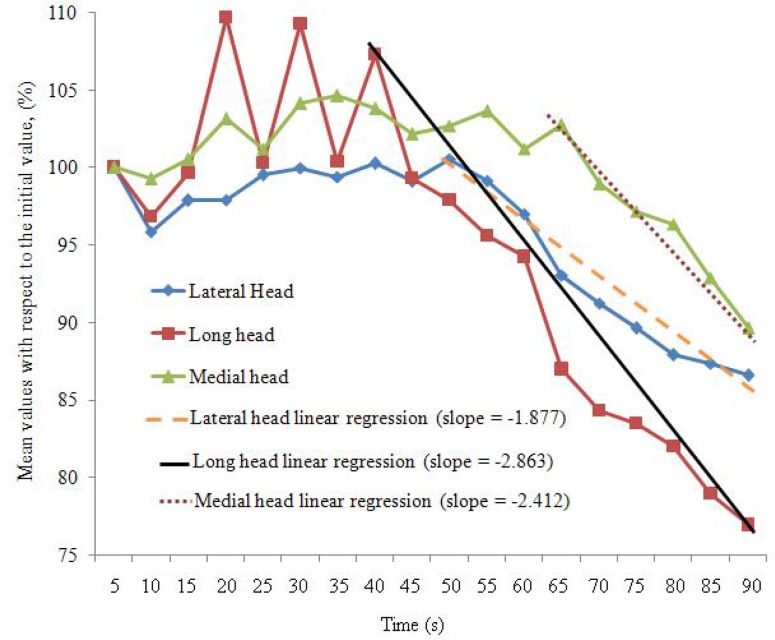
Change in the mean value of the normalized RMS values (expressed as a percentage of the initial value) obtained for the three heads of the TB during the 90 s period of isometric contraction

Descriptive statistics for the mean, SD and CV% values of all subjects are presented in Table 1. Figure 2 shows the plot of mean values for each period (5 s) expressed as percentages of the initial period (1) values throughout the task duration and the corresponding linear regression slope during fatiguing period for each of the TB heads. Paired t-test significant values between the initial period (1) and the following periods (2–18) for the three heads of TB are presented in Table 2.

## Discussion

We evaluated the changes in the sEMG derived signal variables from the three heads of the TB during a concurrent isometric contraction of a controlled forceful hand grip task and full elbow extension. A number of studies (Bigland-Ritchie et al., 1979; Jones et al., 1979; Moritani et al., 1986; Oda and Moritani, 1995) have reported a time-dependent decrease in RMS values during voluntary contractions. These time-dependent decreases in RMS values were also observed in the present study. In general, we observed a decrease in the percentage of RMS values in all the heads of the TB after a certain period (time to fatigue) during an isometric contraction and the decreasing rates (fatiguing rates) were different among the three heads of the TB.

Previous studies have reported that a greater force exerted during a task can lead to a more rapid appearance of muscle fatigue (Bellemare and Grassino, 1982; Clark and Carter, 1985; Lind and Petrofsky, 1979). In studies of muscle fatigue, the time to fatigue is subjectively determined by maximum possible task duration of each participant (Crenshaw et al., 1997; Dieën et al., 1993; Lowery et al., 2002) or predefined task duration of each participant (Krivickas et al., 1998; Merletti and Roy, 1996; Oda and Kida, 2001). For example, the study of Moritani et al. (1986) considered the time to fatigue to begin when the integrated sEMG or the RMS of the sEMG showed a gradual decrement throughout the muscle fatigue test. Similarly, the study of Oda and Kida (2001) measured sEMG variables (the TB being one of the investigated muscle) during predefined task duration for each participant (62 s of contraction from a maximal concurrent forceful hand grip and elbow flexion or extension) and identified the time to fatigue of the muscles by analysis of the percentage in the RMS amplitude in every 5 s period of the total contraction based on the initial (1–5 s) period. The authors of this study considered the fatiguing start time to begin when the percentage of RMS amplitudes gradually decreased during an isometric MVC of the muscles.

Following this aforementioned guide, we observed that the percentage RMS values based on the initial period started to decrease at different times among the three heads of the TB during the concurrent isometric contraction of a forceful hand grip for 90 s at 80% MVC and full elbow extension. It was observed that the fatiguing time started at 50 s, 40 s and 65 s in lateral, long and medial heads of TB muscle, respectively. The studies of González-Izal et al. (2010a) and González-Izal et al. (2010b) highly recommended that a linear regression technique should be used to estimate muscle fatigue, which relates changes in sEMG variables to change in power loss (as a direct measurement of muscle fatigue). In the present study, we analysed the RMS values using linear regression and found that the rate of change of this parameter was obtained as follows: the long head with slope = −2.863, followed by the medial head with slope = −2.412 and finally the lateral head with slope = −1.877. These variations in the fatiguing time and fatiguing rates among the heads of the TB are most likely related to the dissimilarity of activity in the heads of TB (Ali et al., 2014) which differ depending on the muscle fibre type and the motor unit recruitment pattern (Gerdle et al., 1991).

## Conclusion

Our findings indicate that fatigue was induced at different times and muscle activity decreased at different rates, among the three heads of TB. These outcomes were observed during a concurrent isometric contraction of a forceful hand grip task for 90 s at 80% MVC and full elbow extension. There is however a possibility that repetitive excessive fatigue of long duration may lead to muscle damage. Thus, increasing TB muscle strength through repetitive resistive training should consider duration, frequency and intensity of the training program. Furthermore, for overall TB strength improvement, through repetitive resistive training, training programs could be designed to systematically target the more susceptible fatiguing heads. Towards achieving these goals, additional studies are required to obtain a better understanding regarding the mechanisms of the TB muscle in order to apply the findings in fields such as physiology, rehabilitation, sports science, and signal processing for prosthesis device control.

## Figures and Tables

**Table 1:** Mean, SD and CV% of the normalized RMS values expressed as percentage for the lateral, long and medial heads of TB of 18 subjects for every 5 s during a total of 90 s contraction period

Contraction		Lateral Head			Long Head			Medial Head		

Period	Seconds	Mean	SD	CV%	Mean	SD	CV%	Mean	SD	CV%
1	01–05	56.93	13.55	23.80	62.44	14.95	23.94	73.76	7.02	9.52
2	06–10	54.57	12.51	22.92	60.45	14.95	24.73	73.22	5.67	7.74
3	11–15	55.72	12.61	22.63	62.21	17.36	27.91	74.12	10.91	14.72
4	16–20	55.75	13.98	25.08	68.47	21.26	31.05	76.06	9.52	12.52
5	21–25	56.65	11.42	20.16	62.61	21.78	34.79	74.63	10.24	13.72
6	22–30	56.92	18.17	31.92	68.22	25.89	37.95	76.79	11.29	14.70
7	31–35	56.58	14.74	26.05	62.69	23.35	37.25	77.17	10.82	14.02
8	36–40	57.07	14.16	24.81	66.98	20.26	30.25	76.56	9.39	12.26
9	41–45	56.41	10.84	19.22	62.00	14.102	22.75	75.34	11.39	15.12
10	46–50	57.23	13.12	22.93	61.14	13.53	22.13	75.72	9.60	12.68
11	51–55	56.44	12.87	22.80	59.68	14.86	24.90	76.44	10.24	13.40
12	56–60	55.22	14.52	26.29	58.84	12.07	20.51	74.66	10.91	14.61
13	61–65	52.98	13.09	24.71	54.31	12.54	23.09	75.77	7.63	10.07
14	66–70	51.94	12.57	24.20	52.64	13.27	25.21	72.96	8.61	11.80
15	76–75	51.03	10.63	20.83	52.12	13.81	26.50	71.68	7.34	10.24
16	76–80	50.04	10.45	20.88	51.20	19.00	37.11	71.07	7.51	10.57
17	81–85	49.75	12.41	24.94	49.29	15.57	31.59	68.51	7.52	10.98
18	86–90	49.29	10.02	20.33	48.05	14.20	29.55	66.11	8.36	12.65

**Table 2:** Paired t-test significant values in the three heads of TB obtained from comparison of periods (initial and other periods)

Between period 1 & …	Significant value (*p*)		

	Lateral head	Long head	Medial head
2	0.14	0.52	0.77
3	0.58	0.96	0.90
4	0.61	0.13	0.32
5	0.87	0.97	0.68
6	0.99	0.28	0.21
7	0.85	0.96	0.11
8	0.95	0.30	0.23
9	0.85	0.91	0.59
10	0.90	0.77	0.42
11	0.97	0.57	0.29
12	0.57	0.39	0.74
13	0.16	0.08	0.35
14	0.09	0.04	0.71
15	0.10	0.07	0.25
16	0.03	0.05	0.19
17	0.03	0.02	0.02
18	0.02	0.01	0.01
